# Molecular characterization and expression analysis of annexin B3 and B38 as secretory proteins in *Echinococcus granulosus*

**DOI:** 10.1186/s13071-021-04596-7

**Published:** 2021-02-08

**Authors:** Hongyu Song, Xue He, Xiaodi Du, Ruiqi Hua, Jing Xu, Ran He, Yue Xie, Xiaobin Gu, Xuerong Peng, Guangyou Yang

**Affiliations:** 1 Department of Parasitology, College of Veterinary Medicine, Sichuan Agricultural University, Wenjiang, 611130 China; 2 Department of Chemistry, College of Life and Basic Science, Sichuan Agricultural University, Wenjiang, 611130 China

**Keywords:** *Echinococcus granulosus*, Annexin, Calcium ion, Western blotting, Immunofluorescence localization, Secretory protein

## Abstract

**Background:**

Cystic echinococcosis is a parasitic zoonotic disease, which poses a threat to public health and animal husbandry, and causes significant economic losses. Annexins are a family of phospholipid-binding proteins with calcium ion-binding activity, which have many functions.

**Methods:**

Two annexin protein family genes [*Echinococcus granulosus* annexin B3 (*Eg*AnxB3) and *Eg*AnxB38] were cloned and molecularly characterized using bioinformatic analysis. The immunoreactivity of recombinant *Eg*AnxB3 (r*Eg*AnxB3) and r*Eg*AnxB38 was investigated using western blotting. The distribution of *Eg*AnxB3 and *Eg*AnxB38 in protoscoleces (PSCs), the germinal layer, 18-day strobilated worms and 45-day adult worms was analyzed by immunofluorescence localization, and their secretory characteristics were analyzed preliminarily; in addition, quantitative real-time reverse transcription polymerase chain reaction was used to analyze their transcript levels in PSCs and 28-day strobilated worms stages. The phospholipid-binding activities of r*Eg*AnxB3 and r*Eg*AnxB38 were also analyzed.

**Results:**

*Eg*AnxB3 and *Eg*AnxB38 are conserved and contain calcium-binding sites. Both r*Eg*AnxB3 and r*Eg*AnxB38 could be specifically recognized by the serum samples from *E*. *granulosus*-infected sheep, indicating that they had strong immunoreactivity. *Eg*AnxB3 and *Eg*AnxB38 were distributed in all stages of *E*. *granulosus*, and their transcript levels were high in the 28-day strobilated worms. They were found in liver tissues near the cysts. In addition, r*Eg*AnxB3 has Ca^2+^-dependent phospholipid-binding properties.

**Conclusions:**

*Eg*AnxB3 and *Eg*AnxB38 contain calcium-binding sites, and r*Eg*AnxB3 has Ca^2+^-dependent phospholipid-binding properties. *Eg*AnxB3 and *Eg*AnxB38 were transcribed in PSCs and 28-day strobilated worms. They were expressed in all stages of *E*. *granulosus*, and distributed in the liver tissues near the hydatid cyst, indicating that they are secreted proteins that play a crucial role in the development of *E*. *granulosus*.

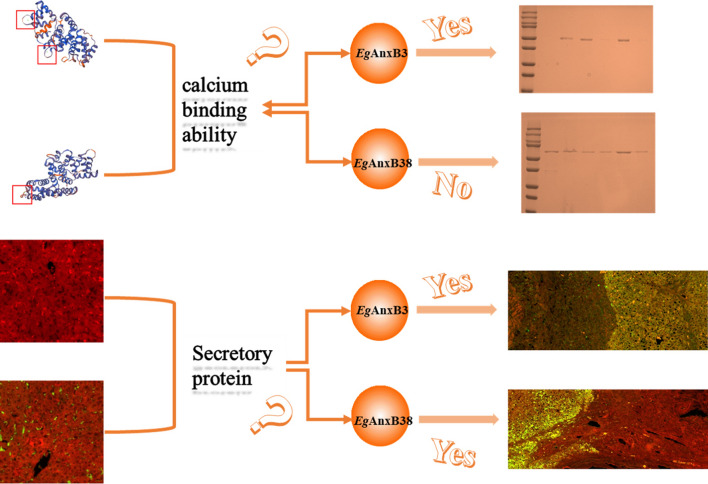

## Background

Cystic echinococcosis (CE), which is caused by the larval stage of *Echinococcus granulosus*, is a parasitic zoonosis currently prevalent in Mediterranean countries, southern South America, Central Asia, China, Australia and parts of Africa [1, 2]. CE seriously threatens public health and impedes the development of animal husbandry, resulting in approximately US $ 3 billion losses to the livestock industry each year [3]. It has been listed as one of the neglected tropical diseases by the World Health Organization [4, 5]. *E*. *granulosus* pathogenic research is of great significance, and studying the mechanism of the interaction between *E*. *granulosus* and its host is also necessary for the prevention and control of CE.

Proteomics analysis revealed that annexins were present in *E*. *granulosus* hydatid fluid, protoscoleces (PSCs) culture fluid and exosomes [6–8]. Annexins are a family of phospholipid-binding proteins with calcium ion-binding activity, which function in calcium signaling, and are widely distributed in eukaryotic cells [9]. According to differences in gene location and protein structure, annexins are divided into five categories [10]. Annexins have multiple functions, such as cell anti-inflammatory activity, membrane repair and membrane transport, and probably participate in cell proliferation, differentiation, and apoptosis [11–15].

Parasite annexins have been found to regulate the host immune response and maintain the structural integrity of the cell [16, 17]. Parasite annexins have also been considered as potential targets for the development of drug and vaccine candidates [16, 18]. *Leishmania* promastigotes could combine annexin V to ensure normal function in the absence of phosphatidylserine [19]. Annexin 2 of *Schistosoma mansoni* is involved in epidermal development [20]. Recombinant annexin B30 of *S*. *mansoni* gave no significant protection against the parasite, which suggested it may not be suitable as a vaccine candidate [21]. Annexin B1 of *Taenia solium* can downregulate the immune response of the host [22, 23]. In humans, *E. granulosus* annexins have only been found in the fluid of pulmonary hydatids, not in the fluid of vertebral or paravertebral hydatids, which suggests that they play an important role in the calcification process of cysts [7]. Up until now, only *E. granulosus* annexin B33 (*Eg*AnxB33) has been studied [24]. In the present study, cloning, expression, bioinformatic analysis, western blotting, relative fluorescence quantitative polymerase chain reaction (PCR), immunofluorescence localization, and phospholipid–binding bioactivity analyses of *Eg*AnxB3 and *Eg*AnxB38 were performed to provide basic information and new research directions for the interaction between *E*. *granulosus* and its hosts.

## Methods

### Animals

Four female New Zealand rabbits (9 weeks old) were purchased from Dashuo Experimental Animal (Chengdu, China; experimental animal production license no. SYXK2019-189). Four male beagles (6 months old) were provided by the Dujiangyan Beagle Breeding Center of the Sichuan Institute of Musk Deer Breeding. Albendazole and levamisole were used for deworming in the first 3 months.

### Parasites

Cysts of *E*. *granulosus* were obtained from naturally infected sheep in Sichuan Province, China. PSCs and the germinal layer were separated aseptically as described previously [25, 26]. The 28-day strobilated worms were acquired by artificially infecting beagles. Each beagle was given 50,000 PSCs orally and then euthanized after 28 days. The 18-day strobilated worms and 45-day adult worms were provided by the Department of Parasitology of Sichuan Agricultural University.

### Sera

Sera against *E*. *granulosus* were isolated from naturally infected sheep. Negative sera were collected from cestode-free sheep. Corresponding sera were obtained in Sichuan Province, China and infection was determined by autopsy.

The preparation process of the polyclonal antibodies of recombinant *Eg*AnxB3 (r*Eg*AnxB3) and r*Eg*AnxB38 was the same as previously reported [26]. Briefly, rabbit sera were collected as the negative control before immunization. Then each rabbit was immunized with 200 μg recombinant protein emulsified with Freund’s complete adjuvant (Sigma-Aldrich, St. Louis, MO), followed by three boosters using Freund’s incomplete adjuvant. Two weeks after the final immunization, antisera were isolated and immunoglobulin G (IgG) was extracted.

### Cloning of *Eg*AnxB3 and *Eg*AnxB38

An RNA extraction kit (Tiangen, Beijing, China) was used to extract total RNA from PSCs, and the Reverse Transcription System Kit (TaKaRa, Dalian, China) was used to synthesize cDNA. Specific primers were designed at GenBank using the sequences of *Eg*AnxB3 and *Eg*AnxB38 (accession numbers XM_024495323.1 and XM_024494485.1, respectively). The sequence of *Eg*AnxB3 was amplified using the primers 5′-CGGATCCATGGCGACTGTCAAGCCTTGCTG-3′ (BamHI) and 5′-CCGGAATTCTTACTCCAGTATGGCAAGC-3′ (EcoRI). The sequence encoding *Eg*AnxB38 was amplified using primers 5′-CGGATCCATGGCTGGCTATCCTCCACC-3′ (BamHI) and 5′-CGGAATTCTCAGGGTCCAACCAAAGCCAC-3′ (EcoRI). Target fragments were amplified and cloned. Single colonies were selected for PCR identification, and the plasmid from the bacterial solution that tested positive by PCR was sequenced.

### Bioinformatic analysis

The physicochemical properties were predicted using the Expasy proteomics server (http://au.expasy.org). The open reading frames (ORFs) of *Eg*AnxB3 and *Eg*AnxB38 were analyzed using ORF Finder (https://www.ncbi.nlm.nih.gov/orffinder/). Signal peptides and transmembrane area were predicted using online software SignalP (http://www.cbs.dtu.dk/services/SignalP-3.0/) and TMHMM-2.0 (http://www.cbs.dtu.dk/services/TMHMM-2.0/). Tertiary (three-dimensional) structures were modeled through SWISS-MODEL (http://swissmodel.expasy.org/). MEGA software (version 5.05) was used to construct the phylogenetic tree using the maximum likelihood method [27].

### Expression and purification of recombinant *Eg*AnxB3 and *Eg*AnxB38

The correctly sequenced *Eg*AnxB3 and *Eg*AnxB38 plasmids were digested with restriction enzymes, ligated into the pET32a(+) plasmid. The resulting recombinant plasmids were transformed into *Escherichia coli* BL21 (DE3) (Tiangen, Beijing). *E. coli* cells containing pET32a-*Eg*AnxB3 and pET32a-*Eg*AnxB38 were cultivated at 37 °C for 8 h. Then the transformants were induced with 1 mM isopropyl β-D-1-thiogalactopyranoside. The recombinant proteins were purified using a Ni^2+^ affinity chromatography column (Bio-Rad, Hercules, CA).

### Western blotting

Western blotting was performed as described previously [28]. Briefly, the crude protein extracts of PSCs and the purified recombinant protein were transferred onto a polyvinylidene difluoride membrane. The membrane was incubated with sera [1:200 volume/volume (v/v) dilution] from CE-infected sheep or polyclonal antibodies (1:200 v/v dilution) for 12 h at 4 °C. After four washes, horseradish peroxidase (HRP)-conjugated sheep anti-rabbit IgG or rabbit anti-sheep IgG (1:2000 v/v dilution; Boster, Wuhan, China) was added and incubated for 1 h at 37 °C. The immunoreactive protein signals were visualized using an Enhanced HRP-DAB Chromogenic Substrate Kit (Tiangen).

### Quantitative real-time reverse transcription PCR

Total RNA and cDNA of PSCs and 28-day strobilated worms were obtained as described above. Quantitative real-time reverse transcription PCR was used to analyze expression profiles of *EgAnxB3* and *EgAnxB38* in PSCs and 28-day strobilated worms. The primers for *Eg*AnxB3 were 5′-TGCCAACACGGATGCCCAAAC-3′ and 5′-CTGGTGCGGTGTGCGAGAAC-3′. The primers for *Eg*AnxB38 were 5′-CGCTACGCAGAGGACAAGAACG-3′ and 5′-CTCGCATCTACCCAGCACCAAC-3′. Expression of the actin gene was detected for use as an internal control for normalization. Primers specific to *E. granulosus* actin were 5′-ATGGTTGGTATGGGACAAAAGG-3′ and 5′-TTCGTCACAATACCGTGCTC-3′. The data were analyzed using the 2^−ΔΔCT^ method [29].

### Immunofluorescence localization

Immunofluorescence localization was performed as described previously [26]. The sections (PSCs, germinal layer from fertile/infertile cysts, 18-day strobilated worms and 45-day adult worms) were incubated with purified anti-r*Eg*AnxB3/anti-r*Eg*AnxB38 rabbit IgG or negative rabbit sera (1:200 v/v dilutions) for about 14 h at 4 °C. Fluorescein isothiocyanate-conjugated sheep anti-rabbit IgG (1:100 dilution in 0.1% Evans blue solution) was incubated with sections for 1 h at 37 °C in the dark. The fluorescence signals were observed under a fluorescence microscope. Meanwhile, to detect the possible secretion of *Eg*AnxB3 and *Eg*AnxB38, the liver tissues away from the cysts and near the cyst wall were also evaluated [24].

### Phospholipid-binding bioactivity assay

The preparation of liposomes was based on previous reports with some modifications [24, 30]. Soybean lecithin (0.9 g; Sangon, Shanghai, China) and 0.3 g cholesterol (Sangon) were mixed and dissolved in anhydrous ethanol in a small beaker; the beaker was placed in a 65–70 °C water bath with stirring in order that the contents could dissolve completely, after which the beaker was rotated to remove the ethanol. Thirty milliliters of preheated phosphate-buffered saline was added to the beaker containing lecithin and cholesterol lipid membrane, and the contents stirred and heated in a water bath at 65–70 °C for 10 min. Finally, the beaker was placed on a magnetic stirrer and the contents stirred for 30–60 min. The phospholipid-binding assay was performed as described previously [24, 31]. There were three experimental groups for each recombinant protein: A, B, and C. Twenty microliters of liposomes, 30 μl r*Eg*AnxB3/r*Eg*AnxB38, and 30 μl of 1 mM CaCl_2_ (except for group C) was added to each group, and 50 mM Tris–HCl added as a supplement to a total volume of 100 μl. All the groups were incubated in ice water and centrifuged to separate the supernatant from the precipitate. The precipitate in group B was washed with Tris–HCl. Thirty microliters of 1 mM ethylenediaminetetraacetic acid (EDTA) and 70 μl of Tris–HCl was added to the precipitate of group B and the mixture incubated in ice water for 30 min. The supernatant and precipitate were separated by centrifugation. All the supernatant and precipitate samples were analyzed using 12% sodium dodecyl sulphate–polyacrylamide gel electrophoresis.

## Results

### Gene amplification and bioinformatics analysis

The results of the bioinformatics analysis of *Eg*AnxB3 and *Eg*AnxB38 are shown in Table 1. Both *Eg*AnxB3 and *Eg*AnxB38 were predicted to be extracellular proteins, but without a signal peptide.

**Table 1:** Bioinformatics analysis of *Echinococcus granulosus* annexin B3 (*Eg*AnxB3) and *Eg*AnxB38

	Open reading frame	Amino acid	Molecular weight (kDa)	Isoelectric point (pI)	Lipid solubility coefficient	Instability coefficient
*Eg*AnxB3	933	310	34.8	5.37	82.55	33.45
*Eg*AnxB38	1356	451	48.5	6.66	68.63	35.46

**Fig. 1:**
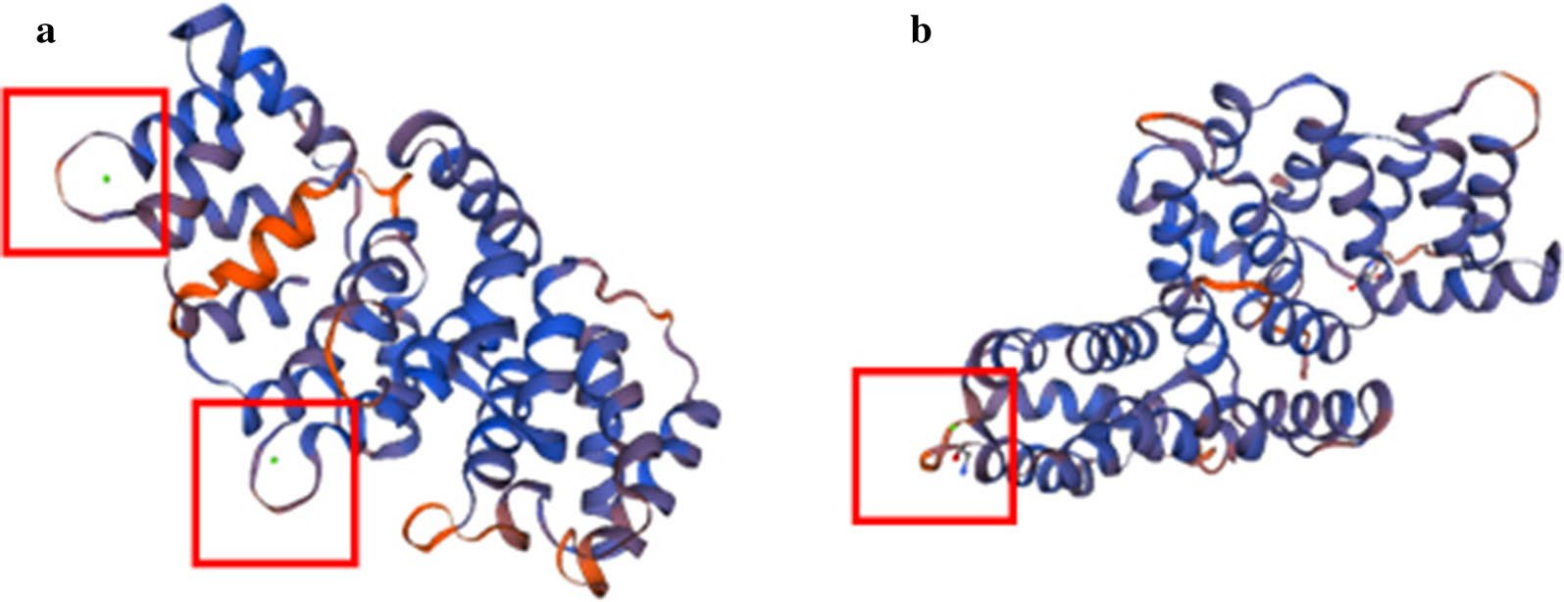
Three-dimensional structure model prediction of *Echinococcus granulosus* annexin B3 (*Eg*AnxB3) (**a**) and *Eg*AnxB38 (**b**). The* red box* shows the calcium ion-binding area

Homology modeling was carried out on *Eg*AnxB3 and *Eg*AnxB38 in the SWISS-MODEL database, and two proteins with high homology were found, whose Protein Data Bank numbers were 1ala.1.A and 1aii.1.A. Using 1ala.1.A and 1aii.1.A as templates, three-dimensional models of *Eg*AnxB3 and *Eg*AnxB38 were constructed, respectively. The similarity between *Eg*AnxB3 and the template sequence was 45.75%, while the similarity was 39.31% between *Eg*AnxB38 and the template sequence. Both *Eg*AnxB3 and *Eg*AnxB38 have calcium-binding sites, but *Eg*AnxB3 contains two types of calcium ion-binding domain, whereas *Eg*AnxB38 only contains one calcium ion-binding domain (Fig. 1).

**Fig. 2:**
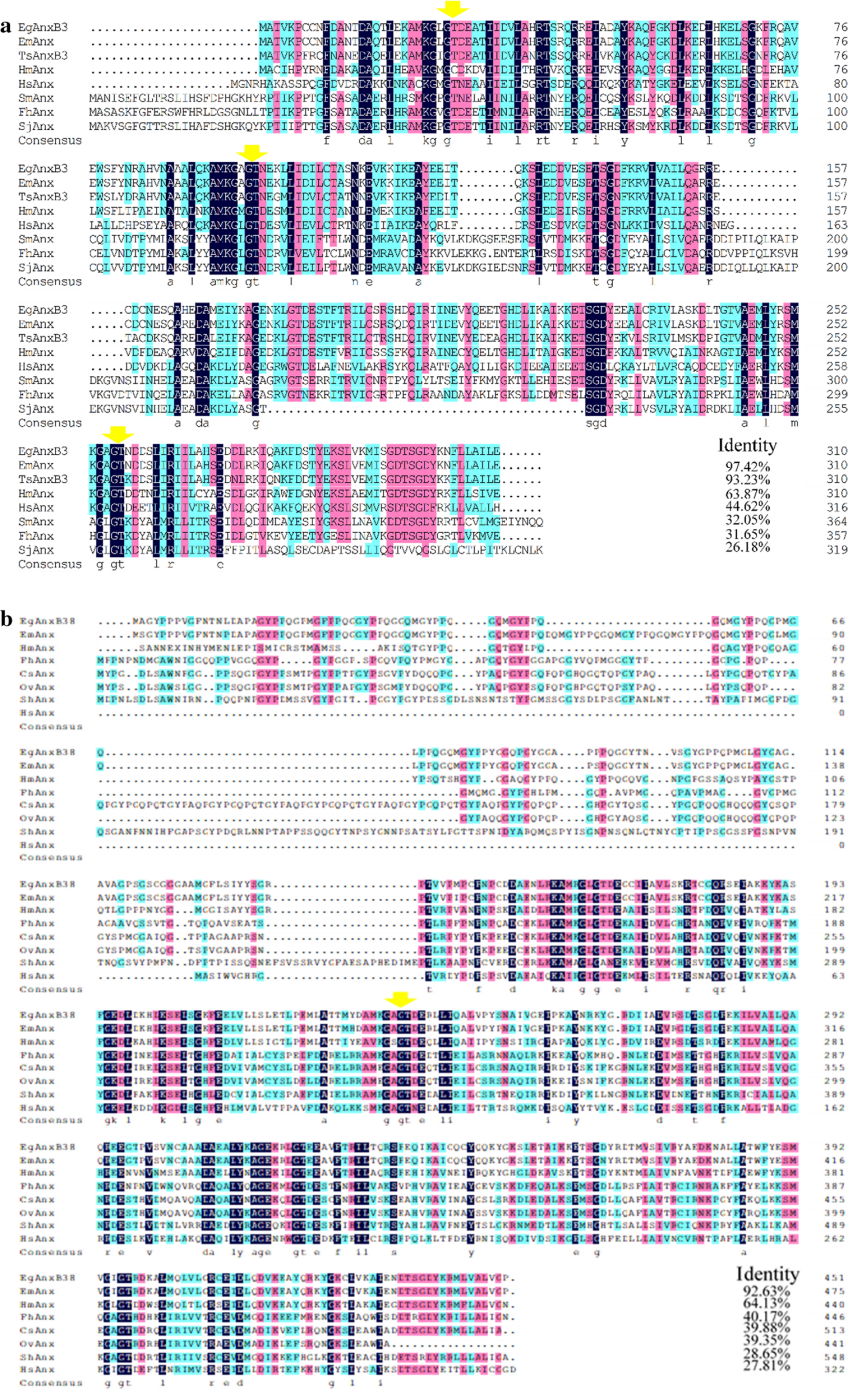
Sequence alignment of *Eg*AnxB3 (**a**) and *Eg*AnxB38 (**b**). Note the K-G-X-G-T sequence, indicated by* yellow arrows*. **a*** Eg* *Echinococcus granulosus* [GenBank (gb) XP_024350278.1],* Em* *Echinococcus multilocularis* (gb CDI98572.1),* Ts* *Taenia solium* (gb AAY27744.1),* Hm* *Hymenolepis microstoma* (gb CDS27549.1),* Hs* *Homo sapiens* (gb CAG46637.1),* Sm* *Schistosoma mansoni* (gb AAC79802.3),* Fh* *Fasciola hepatica* (gb THD28189.1),* Sj* *Schistosoma japonicum* (gb TNN13360.1). **b*** Eg* *E. granulosus* (gb CDS24484.1),* Em* *E. multilocularis* (gb CDS35601.1),* Hm* *Hymenolepis microstoma* (gb CDS26802.2),* Fh* *F. hepatica* (gb THD25992.1),* Cs* *Clonorchis sinensis* (gb RJW68544.1),* Ov* *Opisthorchis viverrini* (gb OON18674.1),* Sh* *Schistosoma haematobium* (gb XP_012796077.1),* Hs* *Homo sapiens* (gb AAH00871.1); for other abbreviations, see Fig. 1

**Fig. 3:**
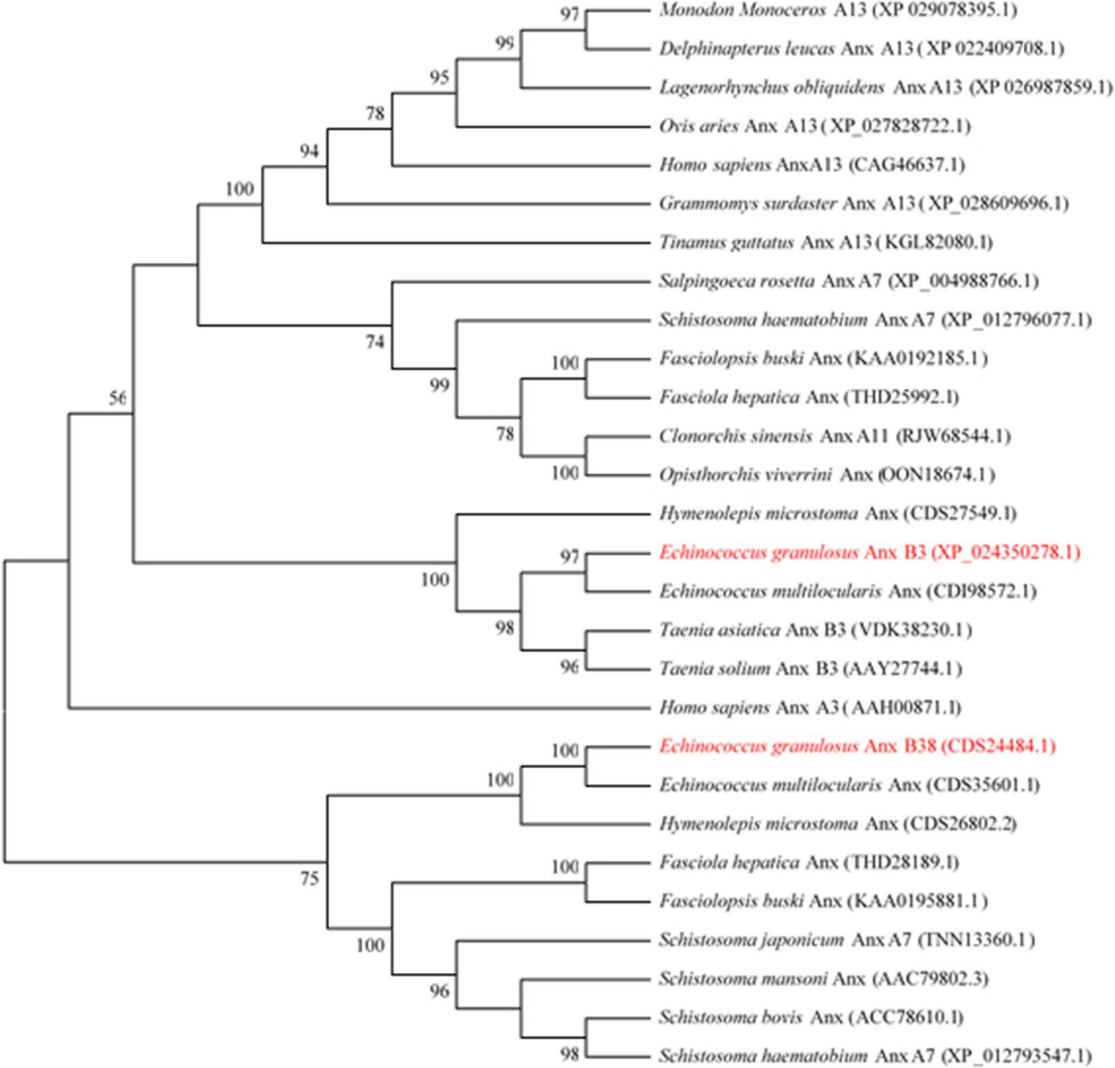
Maximum likelihood phylogenetic tree of *Eg*AnxB3 and *Eg*AnxB38. For abbreviations, see Fig. 1

DNAMAN was used to compare the protein sequences of *Eg*AnxB3 and *Eg*AnxB38, and it was found that the similarity was 32.15%. Multiple sequence alignment revealed that *Eg*AnxB3 shared 97.42% identity with *Echinococcus multilocularis* annexin, 83.23% with *T. solium* AnxB3, and 63.87% with *Hymenolepis microstoma* annexin (Fig. 2a). *Eg*AnxB38 shared 92.63% identity with *E. multilocularis* annexin, 64.13% with *H. microstoma* annexin, 40.17% with *Fasciola hepatica* annexin, and 39.88% with *Clonorchis sinensis* annexin (Fig. 2b). The maximum likelihood phylogenetic tree demonstrated that *Eg*AnxB3 and *Eg*AnxB38 were located on different branches, and had the closest genetic relationship with *E*. *multilocularis* (Fig. 3).

### Expression, purification, and western blotting of r*Eg*AnxB3 and r*Eg*AnxB38

**Fig. 4:**
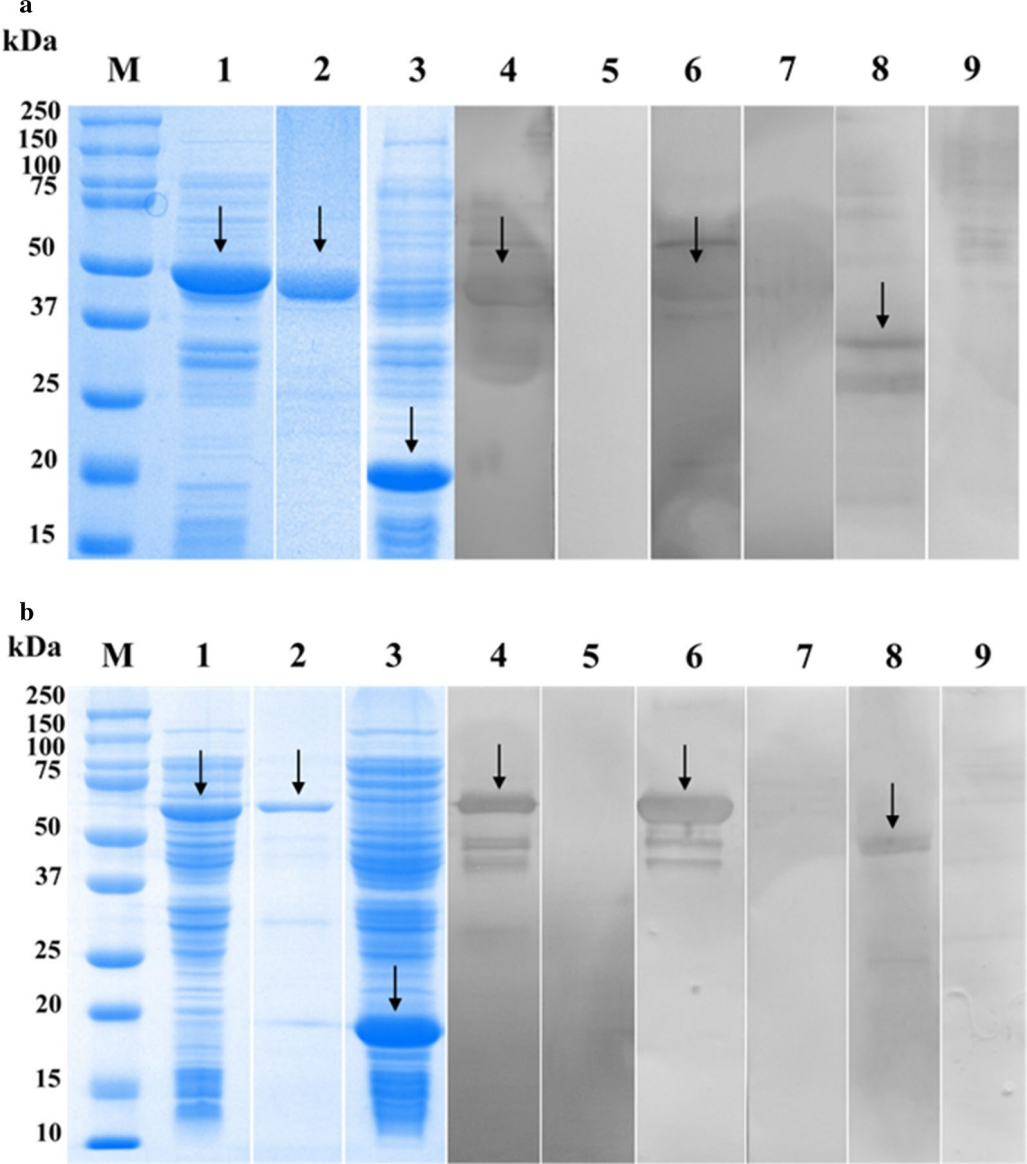
Purification and western blotting of recombinant *Eg*AnxB3 (r*Eg*AnxB3) (**a**) and r*Eg*AnxB38 (**b**).* Lane M* Protein marker,* lane 1* r*Eg*AnxB3/r*Eg*AnxB38 crude protein,* lane 2* purified r*Eg*AnxB3/r*Eg*AnxB38,* lane 3* pET32a(+) induced by isopropyl β-D-1-thiogalactopyranoside,* lane 4* r*Eg*AnxB3/r*Eg*AnxB38 incubated with rabbit anti-r*Eg*AnxB3/r*Eg*AnxB38-immunoglobulin G (IgG),* lane 5* r*Eg*AnxB3/r*Eg*AnxB38 incubated with negative rabbit serum,* lane 6* r*Eg*AnxB3/r*Eg*AnxB38 incubated with serum from a sheep infected with *E*. *granulosus*,* lane 7* r*Eg*AnxB3/r*Eg*AnxB38 incubated with serum of a healthy sheep,* lane 8* crude protein of protoscoleces (PSCs) incubated with rabbit anti-r*Eg*AnxB3/r*Eg*AnxB38-IgG,* lane 9* crude protein of PSCs incubated with negative rabbit serum IgG. For other abbreviations, see Fig. 1

The molecular mass of *Eg*AnxB3 was about 52 kDa, which was close to the expected value [note: the pET-32a(+) tag proteins weigh about 18 kDa]. Solubility analysis showed that *Eg*AnxB3 was largely expressed as a soluble form and partly in inclusion bodies (Fig. 4a). The molecular mass of *Eg*AnxB38 was about 67 kDa. Solubility analysis showed that *Eg*AnxB38 was largely expressed in inclusion bodies (Fig. 4b). Western blotting showed that r*Eg*AnxB3 and r*Eg*AnxB38 reacted with rabbit anti-r*Eg*AnxB3 antibody and rabbit anti-r*Eg*AnxB38 antibody, respectively, and with CE-positive sheep sera. Moreover, the native *Eg*AnxB3 protein and *Eg*AnxB38 protein in crude protein from PSCs were recognized using the rabbit anti-r*Eg*AnxB3 antibody and rabbit anti-r*Eg*AnxB38 antibody, respectively (Fig. 4).

### Transcriptional profiles of *Eg*AnxB3 and *Eg*AnxB38

**Fig. 5:**
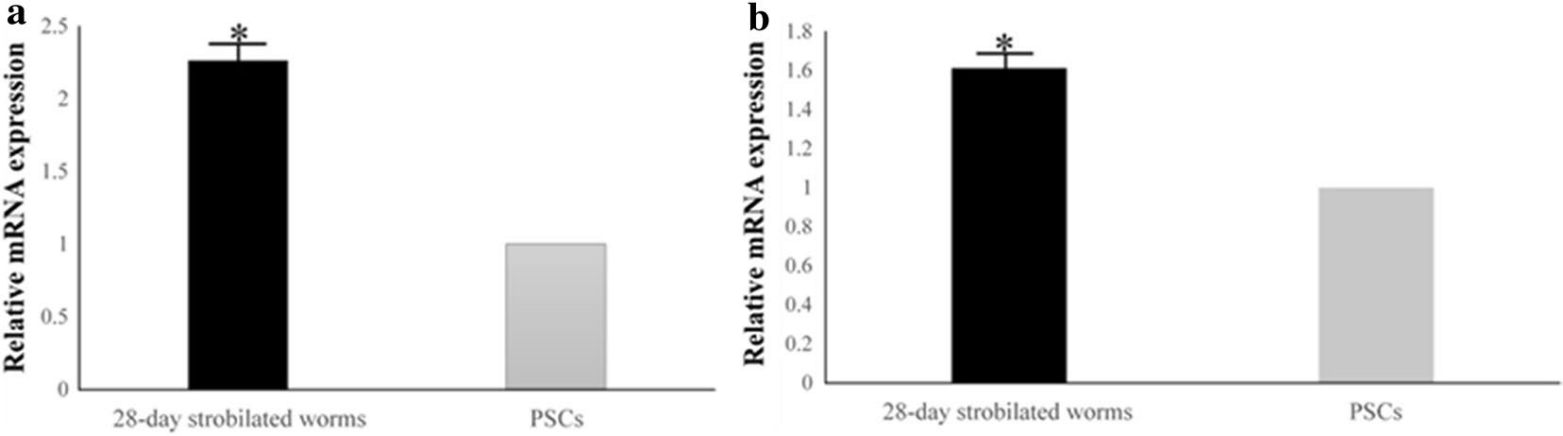
Comparison of transcript levels of *EgAnxB3* (**a**) and *EgAnxB38* (**b**) genes in the PSCs and 28-day strobilated worms. Data are presented as the mean ± SD of triplicate experiments. Statistically significant differences between PSCs (as the control) and 28-day strobilated worms were determined using Student’s* t*-test [*Eg*AnxB3, *t*_(4)_= 14.577, *P *= 0.00013; *Eg*AnxB38, *t*_(4)_= 20.744, *P *= 0.00003] (* P < 0.05). For abbreviations, see Figs. 1 and 4

*EgAnxB3* and *EgAnxB38* genes were transcribed in both PSCs and 28-day strobilated worms, and the transcript levels of *EgAnxB3* and *EgAnxB38* in 28-day strobilated worms were both significantly higher (*P* < 0.05) than those in the PSCs (Fig. 5).

### Immunolocalization of *Eg*AnxB3 and *Eg*AnxB38

**Fig. 6:**
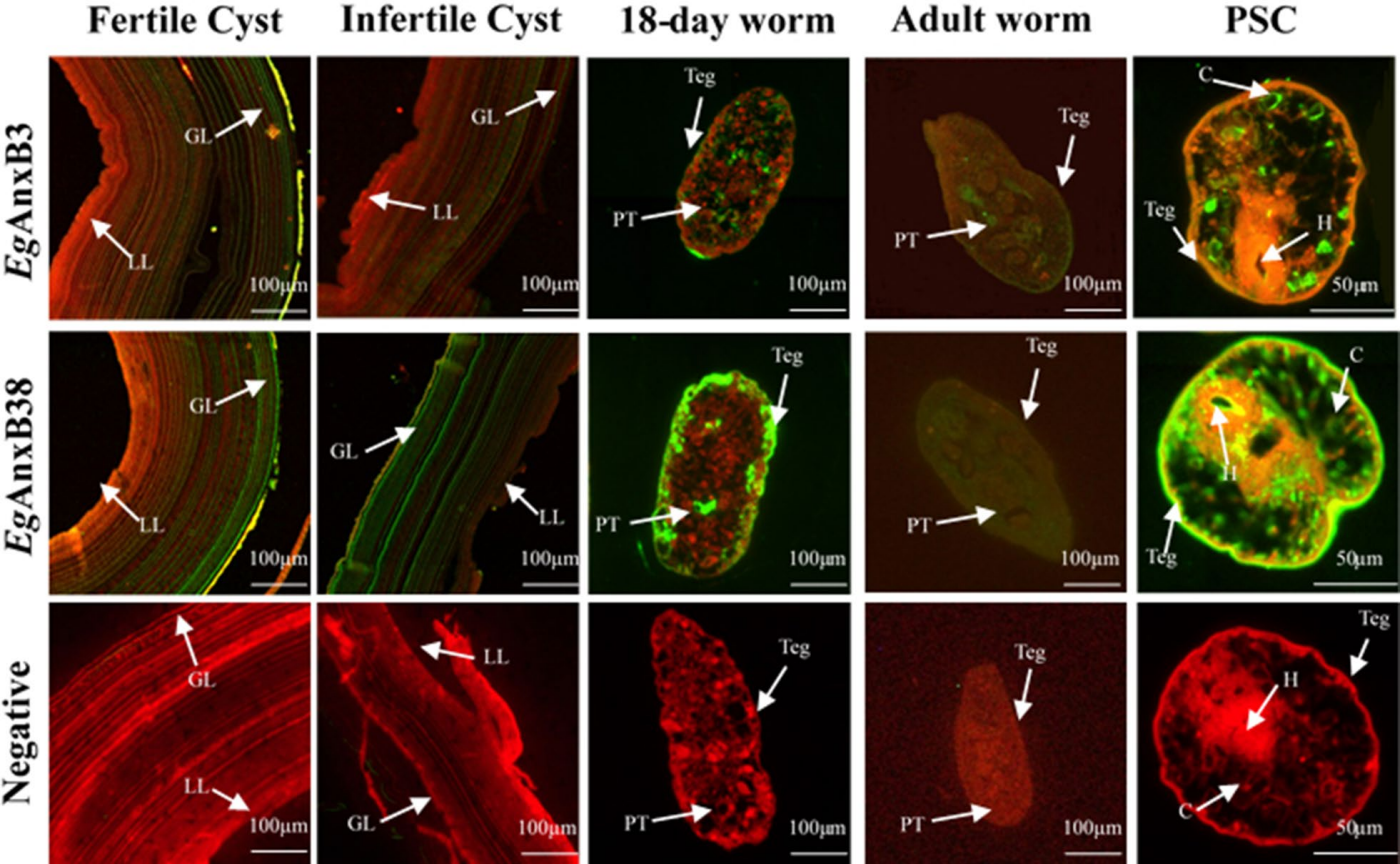
Immunolocalization of *Eg*AnxB3 and *Eg*AnxB38 in different life cycle stages of *E*. *granulosus*. The* green fluorescent region* is the protein distribution region.* GL* germinal layer,* LL* laminated layer,* Teg* tegument,* PT* parenchyma,* H * hooks,* C* calcareous corpuscles; for other abbreviations, see Fig. 1

*Eg*AnxB3 and *Eg*AnxB38 were distributed in the germinal layer of fertile/infertile cysts, the parenchyma and tegument of 18-day strobilated worms and 45-day adult worms, and the calcareous corpuscles and hooks of PSCs. Compared with *Eg*AnxB3, *Eg*AnxB38 was not only distributed in the calcareous corpuscles and hooks of PSCs, but also in the tegument of PSCs. Meanwhile, the distribution range of *Eg*AnxB38 in fertile/infertile cysts, adult worms, and PSCs was significantly wider than that of *Eg*AnxB3, showing a strong fluorescence signal (Fig. 6).

**Fig. 7:**
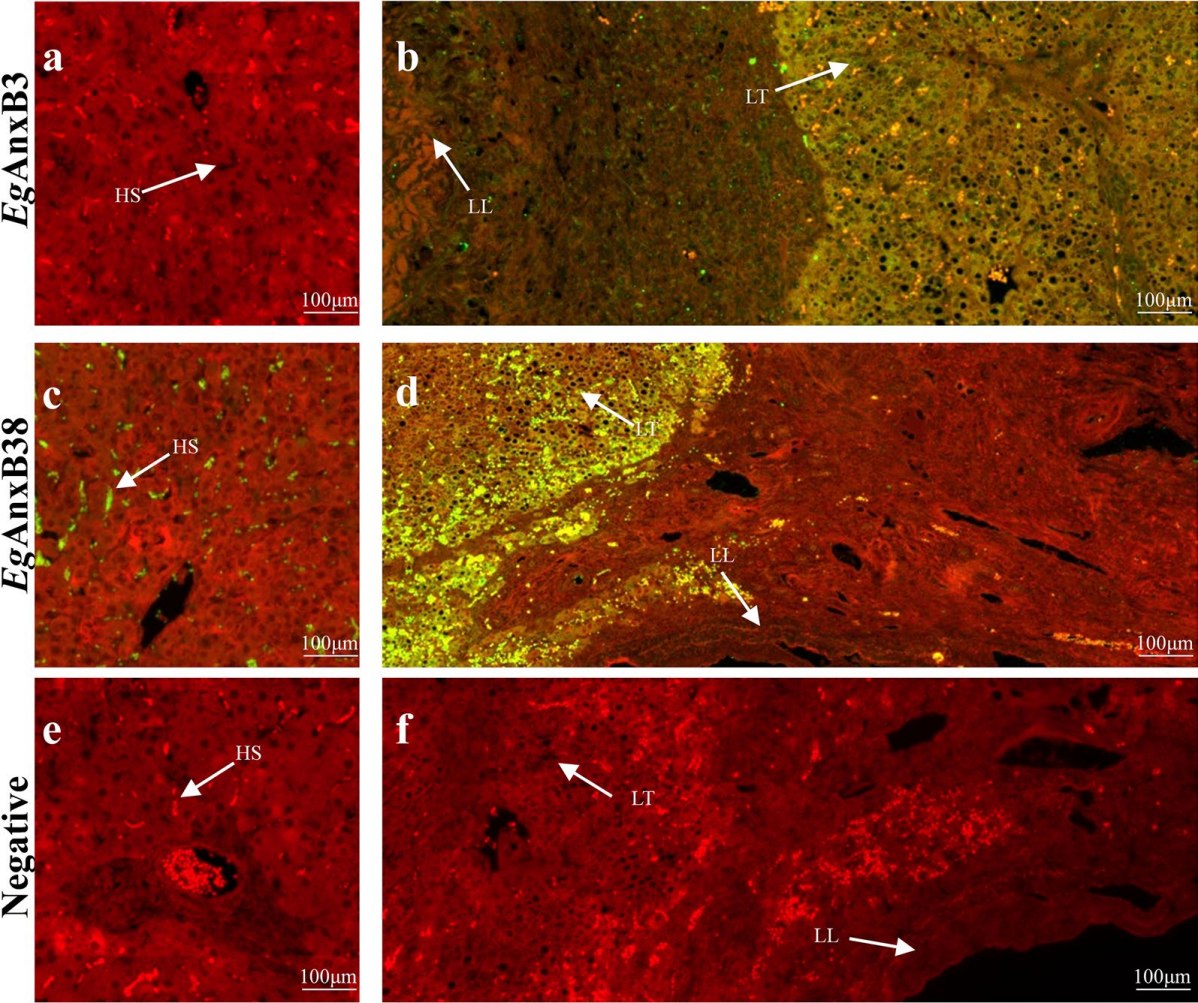
**a**–**f** Immunolocalization of *Eg*AnxB3 and *Eg*AnxB38 in different regions of sheep liver infected with *E. granulosus*. **a**, **c**, **e** Liver tissue distant from the hydatid cysts. **b**, **d**, **f** Liver tissue close to hydatid cysts. The* green fluorescent region* is the protein distribution region.* HS* hepatic sinusoid,* LL* laminated layer,* LT* liver tissue; for other abbreviations, see Fig. 1

*Eg*AnxB3 and *Eg*AnxB38 were distributed in hepatic sinusoids of liver tissue close to hydatid cysts. In addition, *Eg*AnxB38 was also found in hepatic sinusoids in the liver distant from the hydatid cysts, whereas *Eg*AnxB3 was not found in this area (Fig. 7).

### Phospholipid-binding bioactivity analysis

**Fig. 8:**
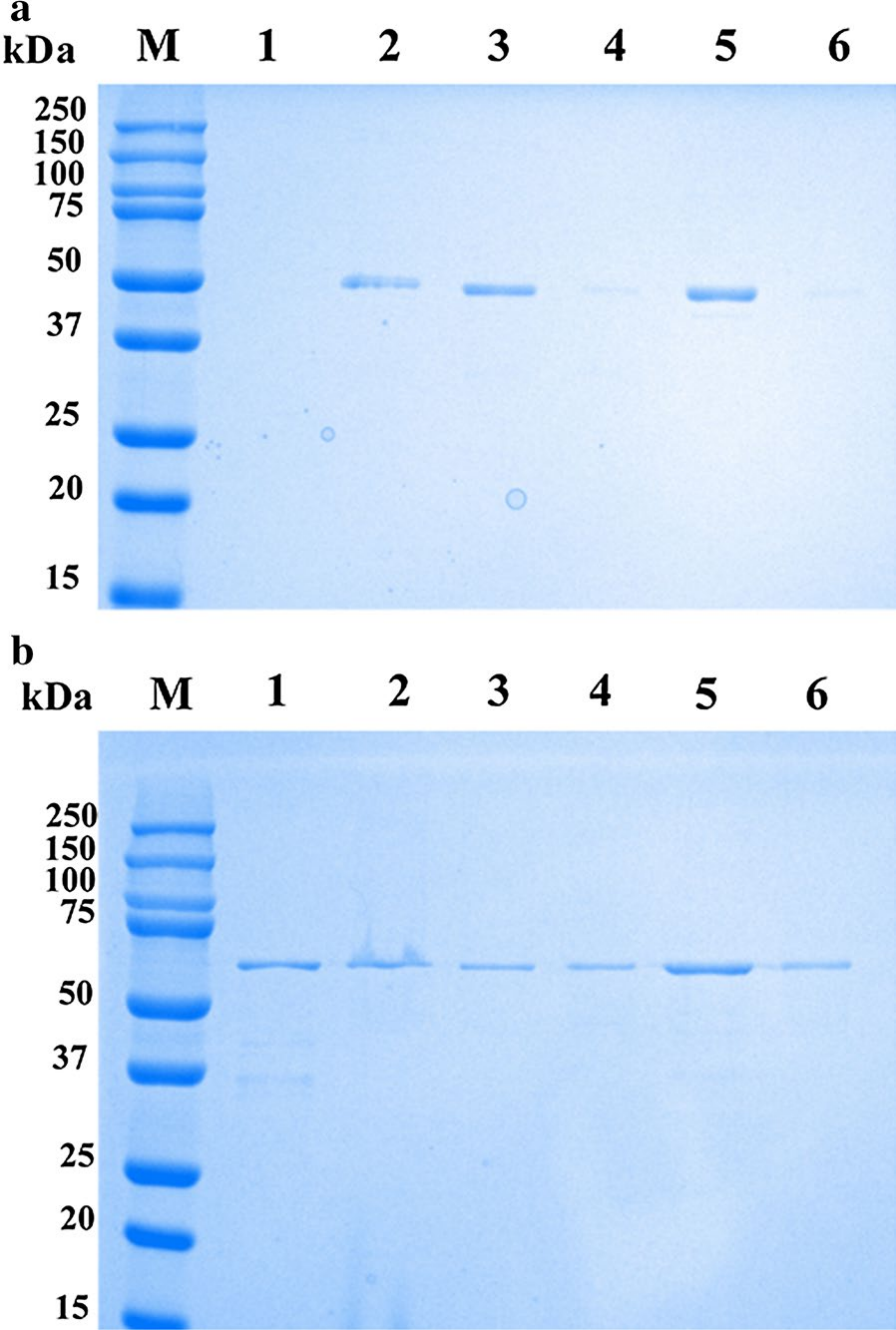
Phospholipid-binding properties of r*Eg*AnxB3 (**a**) and r*Eg*AnxB38 (**b**).* Lanes 1* and* 2* r*Eg*AnxB3/r*Eg*AnxB38 was incubated with liposomes in buffer containing Ca^2+^.* Lanes 3* and* 4* r*Eg*AnxB3/r*Eg*AnxB38 was incubated with liposomes in buffer containing 1 mM Ca^2+^ and 1 mM ethylenediaminetetraacetic acid (EDTA) was then added.* Lanes 5* and* 6* Control group (no Ca^2+^ or EDTA);* lane M* protein marker;* lanes 1*,* 3* and* 5* supernatant;* lanes 2*,* 4* and* 6* precipitate. For other abbreviations, see Fig. 1

Recombinant *Eg*AnxB3 and *Eg*AnxB38 were reacted with lipidosomes in the presence or absence of Ca^2+^ to examine their calcium-dependent phospholipid-binding properties. The mixtures were centrifuged, and the bound protein was pelleted with the precipitate and the unbound protein remained in the supernatant. In the presence of Ca^2+^, r*Eg*AnxB3 was observed in the precipitate rather than in the supernatant (group A). When Ca^2+^ was removed from the recombinant protein using EDTA, r*Eg*AnxB3 largely remained in the supernatant (group B). r*Eg*AnxB3 was mainly observed in the supernatant in the absence of Ca^2+^ (group C). However, r*Eg*AnxB38 was observed in both the precipitate and supernatant, regardless of the presence of Ca^2+^ (Fig. 8).

## Discussion

The classification and nomenclature of annexin B group are confusing. Generally, parasites are invertebrates, and their annexins should be named “annexin B.” However, when we screened *EgAnxB3* and *EgAnxB38* at the National Center for Biotechnology Information based on Zheng’s research in 2013 [32], they were both called “AnxA7,” which is obviously incorrect. We found that *Eg*AnxB3 had high similarity with *T. solium* annexin B3 via sequence alignment, so we named it “*Eg*AnxB3.” However, we did not find an appropriate named parasite sequence with high homology to *Eg*AnxB38. However, we found an article that named the annexins of many parasites by sequence alignment and phylogenetic tree analysis, including the annexins of *E. granulosus* [33]. We searched for *E. granulosus* annexins on the Hofmann Laboratory website (http://www.structuralchemistry.org/annexins/seq/search.php), which showed that our naming of *Eg*AnxB3 is correct, and that the name *Eg*AnxB33 is also consistent with that previously published [24]. Therefore, we confirm here that, for *E. granulosus*, “*Eg*AnxB38” should be used rather than “AnxA7.”

Annexin proteins generally have four homologous repeat domains, and most of the domains contain a typical K–G–X–G–T sequence. It has been reported that the calcium-binding capacity of annexin proteins is based on their K–G–X–G–T sequence [33–35]. The amino acid sequence analysis showed that both *Eg*AnxB3 and *Eg*AnxB38 have four repeat domains and contain a K–X–G–T sequence. The tertiary structure prediction showed that both *Eg*AnxB3 and *Eg*AnxB38 have Ca^2+^-binding sites. To verify the calcium-binding ability of r*Eg*AnxB3 and r*Eg*AnxB38, we conducted a Ca^2+^-dependent phospholipid-binding assay and found that, in the absence of Ca^2+^, r*Eg*AnxB3 could not bind to liposomes and was found in the supernatant, while in the presence of Ca^2+^, r*Eg*AnxB3 bound with liposomes and was found in the precipitate. When EDTA was added to combine with calcium ions, *Eg*AnxB3 was again found in the supernatant, indicating that r*Eg*AnxB3 has Ca^2+^-dependent phospholipid-binding properties. However, the Ca^2+^-dependent phospholipid-binding properties of r*Eg*AnxB38 were relatively weak. r*Eg*AnxB38 was observed in both the precipitate and supernatant, regardless of the presence of Ca^2+^. There are two possible reasons for this. First, in this study, *Eg*AnxB38 was expressed as an inclusion body protein, and thus possibly did not fold correctly, resulting in a lack of calcium-binding capacity. Second, regardless of the amino acid structure analysis or tertiary structure prediction, *Eg*AnxB38 had fewer Ca^2+^-binding sites, resulting in an insufficient calcium-binding capacity.

The transcript levels of annexins are different at various stages of parasite development. *Taenia multiceps AnxB2* and *AnxB12* show high levels of transcription in the oncosphere. The transcript levels of *AnxB2* and *AnxB12* decreased from oncosphere to adult, while that of *AnxB3* increased [34]. The transcript level of *C. sinensis AnxB30* is higher in the metacercaria stage, and lower in adult worms and eggs [36]. The transcript levels of *EgAnxB3 *and *EgAnxB38* in the 28-day strobilated worms were higher than those of PSCs in this study. Considering that annexins can protect the parasite from the host immune system during parasitism, and that PSCs develop into adult worms in the small intestine of the definitive host [16, 17], it is suggested that *Eg*AnxB3 and *Eg*AnxB38 might play a crucial role in the process of PSC invasion of the definitive host.

The parasite communicates with the host through the tegument, and molecules distributed in the tegument participate in the host-parasite interaction, which includes excretion, nutrient absorption, and interaction with the host immune system [37, 38]. In the present study, both *Eg*AnxB3 and *Eg*AnxB38 were found to be located in the tegument and parenchyma of immature and gravid proglottids on the basis of immunofluorescence localization analysis, but *Eg*AnxB38 had a wider distribution and stronger fluorescence intensity. These results indicate that both these proteins might have a paramount role in the process of PSC invasion of the definitive host and development of the parasite, but that *Eg*AnxB38 may possibly play a larger role than *Eg*AnxB3 in this process. *Eg*AnxB38 was distributed in the hooks and tegument of the PSCs, while *Eg*AnxB3 was either not distributed in the hooks and tegument at all, or at a much lower level. Considering that PSCs require the participation of the hook during development in the definitive host’s intestines, it is speculated that *Eg*AnxB38 participates in the interaction between the PSCs and the definitive host. PSCs form and mature in the germinal layer of *E. granulosus* [39]. *Eg*AnxB3 and *Eg*AnxB38 were distributed in the germinal layer, and their distribution range in fertile cysts was larger than that in infertile cysts, suggesting that they might be involved in the growth and development of PSCs.

Most annexins are cytoplasmic proteins or cytoskeletal proteins [40]; however, traces of annexins have also been found in the extracellular regions, although these annexins lack the signal peptide sequences required for extracellular secretion [41–43]. This interesting phenomenon suggests that some annexins are secreted. *C. sinensis* AnxB30 is a secretory protein that is involved in the interaction between the parasite and host, and affects the host’s autoimmune response [36]. As a secreted protein, *T. solium* AnxB1 is involved in the interaction with the host inflammatory cells [16, 22, 23]. Annexins were also found to be present in the excretory–secretory product and hydatid cyst fluid of *E. granulosus* [6, 44]. *Eg*AnxB33 could be detected in the hydatid fluid of *E. granulosus* and was located on the inflammatory cells and fibroblasts of the host-derived layer, suggesting that it might play a paramount role in the interaction between *E*. *granulosus* and the host [24]. Although *Eg*AnxB3 and *Eg*AnxB38 have no signal peptide, in this study, they were found to be located in the hepatic sinusoids of liver tissue near hydatid cysts, indicating that they can be secreted into the extracellular areas to exert their physiological effects through non–classical secretion pathways, such as transfer via extracellular vesicles [45]. *Eg*AnxB38 was also found to be distributed in hepatic sinusoids in the liver, which were far away from the cysts. The fact that *Eg*AnxB38 had a wider distribution in different life cycle stages of *E. granulosus* suggests that it plays a more important role in the development and interaction with the host. Certain excretory or secretory products of the parasite can regulate the host immune system through interaction with the host [46, 47]. The results of this study indicate that further investigation is needed to show if *Eg*AnxB3 and *Eg*AnxB38 play a role as secreted proteins in the immune evasion of *E. granulosus*. In addition, parasite annexins have potential as drug and vaccine targets, so it is also worth exploring if *Eg*AnxB3 and *Eg*AnxB38 can be used as drug targets and vaccine candidates.

## Conclusions

In conclusion, we found that both *Eg*AnxB3 and *Eg*AnxB38 contain calcium–binding sites, and r*Eg*AnxB3 has Ca^2+^-dependent phospholipid-binding properties. Both r*Eg*AnxB3 and r*Eg*AnxB38 could be specifically recognized by CE-positive sheep sera, indicating that they had strong immunoreactivity. The transcription level of *Eg*AnxB3 and *Eg*AnxB38 in 28-day strobilated worms was higher than that in PSCs. *Eg*AnxB3 and *Eg*AnxB38 were distributed in all stages of *E. granulosus*, and were also distributed in the liver tissues near hydatid cysts, indicating that they are secreted proteins that might play a crucial role in the development of *E. granulosus*.

## Data Availability

The datasets supporting the conclusions of this article are included within the article.

## Abbreviations

r*Eg*AnxB3: Recombinant *Echinococcus granulosus* annexin B3
r*Eg*AnxB38: Recombinant *Echinococcus granulosus* annexin B38
PSCs: Protoscoleces
CE: Cystic echinococcosis
ORF: Open reading frame
HRP: Horseradish peroxidase
PCR: Polymerase chain reaction
IgG: Immunoglobulin G
EDTA: Ethylenediaminetetraacetic acid
